# Effects of Diabetes Knowledge and Attitudes Toward Internet Health Information on e-Health Literacy in Middle-Aged Patients with Diabetes

**DOI:** 10.3390/healthcare13050512

**Published:** 2025-02-27

**Authors:** Minsung Lee, Jaelan Shim

**Affiliations:** College of Nursing, Dongguk University WISE, Gyeongju 38066, Republic of Korea; minslee24@gmail.com

**Keywords:** diabetes, e-health literacy, middle-aged, attitudes towards internet health information, diabetes knowledge

## Abstract

**Background/Objectives**: Effective diabetes self-management requires useful knowledge and health-related literacy based on a positive attitude toward seeking health information on the Internet. Therefore, this study aimed to determine the relationship of e-health literacy (eHL) with diabetes knowledge and attitudes toward internet health information in middle-aged patients with diabetes and to identify factors influencing patient eHL. **Methods**: This descriptive correlational study included 185 middle-aged patients with diabetes being followed-up with in the outpatient endocrinology department at a South Korean university hospital between 14 January and 29 February 2024. Data were collected using self-report structured questionnaires and were analyzed using IBM SPSS 27.0. **Results**: eHL was positively correlated with diabetes knowledge (*r* = 0.31, *p* < 0.001) and attitudes toward internet health information (*r* = 0.62, *p* < 0.001). Diabetes knowledge was also positively correlated with attitudes toward internet health information (*r* = 0.25, *p* < 0.001). Attitudes toward internet health information (β = 0.55, *p* = <0.001), diabetes knowledge (β = 0.13, *p* = 0.040), and drinking status (β = 0.12, *p* = 0.044) were significant variables affecting eHL. **Conclusions**: The most influencing factor in eHL in middle-aged patients with diabetes is the patient’s attitudes toward internet health information; diabetes knowledge was identified as a significant predictor. To improve eHL in middle-aged patients with diabetes, it is important to develop nursing intervention strategies to help promote diabetes knowledge and positive attitudes toward internet health information. Healthcare providers should continuously monitor patients to ensure they acquire and utilize correct information.

## 1. Introduction

Diabetes is a chronic disease caused by a disorder of blood glucose control following the failure of the pancreas to produce enough insulin or when the body is unable to use it effectively [1]. Approximately 530 million adults aged 20–79 years are diagnosed with diabetes globally [2], and this number is expected to increase to more than 780 million by 2045, a 47% increase from the current prevalence, affecting one in eight people worldwide [3]. In the case of South Korea, the prevalence of diabetes among adults aged 19 years and older was reported to be 12.5% in 2022. Among such individuals, only 24.2% achieved adequate glycemic control, highlighting the challenges in effective diabetes management [4]. Previous studies showed that it takes 3–5.2 years on average to develop complications after diagnosis with diabetes, and about 12% of patients with diabetes already had complications at the time of diagnosis [5]. Therefore, it is necessary to develop intervention strategies for self-management of diabetes, including glycemic control and prevention of complications from the early stage of diagnosis.

During midlife, people are socially and economically active [6]. It is a period of psychological and social growth as individuals face various life challenges [7] and is also a time when health concerns arise as aging begins [8]. The United States defines middle age as 45–64 years, with the estimated diagnosis rate of diabetes and prediabetes being second only to that in the elderly population aged 65 years and older [9]. According to the analysis of the age group utilizing healthcare institutions for diabetes using the data from the National Health Insurance Service of South Korea (2021) [10], middle-aged adults aged 40–64 years accounted for 48.7%, the highest among all age groups. Therefore, it is essential to provide patients of this age with education and management interventions to prevent chronic diseases [11,12], and the acquisition of disease-related knowledge is critical for such management interventions.

Diabetes knowledge refers to the knowledge that patients with diabetes have about the causes, symptoms, and guidelines for the treatment and management of diabetes [13]. Previous studies revealed that diabetes knowledge influenced complication reduction and treatment compliance [14] and that lower diabetes knowledge was associated with lower levels of health promotion behaviors and higher readmission rates, demonstrating the importance of diabetes knowledge [15]. In a previous study, higher disease-related knowledge, such as diabetes knowledge, was associated with an increase in health information-seeking behavior [16]. In South Korea, previous studies showed that the diabetes knowledge of rural elderly people was significantly higher than that of urban elderly people [17], confirming a relationship between the actual level of diabetes knowledge and health information-seeking behavior is necessary.

Patients with diabetes mainly acquire health information through conversations with healthcare providers, health promotion videos, the internet, and books [18]. Previous studies reported that 64.5% of the 2903 patients with diabetes in the U.S. [19], 58.5% of the 2895 patients with diabetes in Singapore [18], and 27.9% of the 344 patients with diabetes in Saudi Arabia [20] used the Internet to seek health information. Therefore, it is important to acquire useful information by discerning correct information among the vast amount of information available on the internet.

Attitudes toward internet health information refer to the usefulness, ease of use, reliability, and utilization of information as perceived by internet health information seekers [21,22]. In modern society, the internet has become an important source of information. However, the uncertain quality of information available on the internet causes negative emotions, such as anxiety [23], and negatively affects health-related decision-making [24]. Meanwhile, positive attitudes toward internet health information were identified as an important predictor of e-health literacy (eHL) by enhancing health behavior intention and health information-seeking behavior [21,22,25]. Thus, the effective management of chronic diseases requires eHL to obtain appropriate information and critically evaluate and utilize it through positive health information-seeking attitudes.

eHL means the ability to seek, explore, understand, and evaluate health information online, and the capacity to apply and disseminate knowledge to solve health problems [26]. Previous studies reported that eHL was associated with participation in online health and local communities to obtain or contribute information [27] and facilitate communication with healthcare providers [28], positively influencing health outcomes. However, health information from the internet may be based on unclear evidence, be biased, or be of low quality. Therefore, it is important to improve eHL to discern quality information from the vast amount of health information available on the internet and apply it to patients’ decision-making [29].

Despite the importance of eHL, few studies have focused on eHL in patients with diabetes. Most previous studies on eHL of patients with diabetes focus on the entire adult diabetic population [30,31] or the elderly [32,33], whereas the eHL of middle-aged adult patients with diabetes remains understudied.

Thus, in this study, we aimed to examine the level of diabetes knowledge, attitudes toward internet health information, and eHL in middle-aged patients with diabetes and the relationships between these variables and to identify the effect of diabetes knowledge and attitudes toward internet health information on eHL in middle-aged patients.

## 2. Materials and Methods

### 2.1. Study Design and Participants

This descriptive correlational study was conducted from January to February 2024 at a university hospital located in a rural area of South Korea. The inclusion criteria for this study were as follows: (1) Those who understood the purpose of the study, were able to communicate, and gave written consent to participate in the study; (2) adults aged 40–64 years who were diagnosed with type 2 diabetes by a specialist; (3) those without cognitive decline surveyed via AD8 (Eight-item Interview questionnaire, 0~1 points: normal cognitive function [34]; (4) those who had been diagnosed with diabetes for more than 3 months [35]; and (5) those who provided information to the study and agreed to participate voluntarily. In contrast, we excluded those who had illiteracy or severe diabetes complications and those who were bedridden due to severe diabetes complications.

In addition, the number of participants in this study was determined using the G*Power 3.1.9 program to calculate the appropriate sample size using a significance level of 0.05, power of 0.95, medium effect size of 0.15, and 12 predictor variables for multiple linear regression analysis, which yielded 184 participants. Of the 194 respondents who participated in the survey, 185 valid surveys were used in the final analysis, excluding incomplete responses.

### 2.2. Study Tools

#### 2.2.1. General and Disease-Related Characteristics

General characteristics in this study included sex, age between 40 and 64 years corresponding to middle age according to life-cycle criteria, marital status, employment status, education level, alcohol consumption, and smoking.

Disease-related characteristics included time since diagnosis (in months), non-diabetic chronic complications, experience in diabetes education, experience of using apps or wearable devices for health management, and subjective health status.

#### 2.2.2. Diabetes Knowledge

This study measured diabetes knowledge using a modified version of the diabetes knowledge assessment tool developed by Shim et al. [13]. While the original tool consisted of 20 questions, the modified tool had 23 questions, including three questions regarding insulin among the subfactors. The subfactors included six domains: general knowledge (six questions), treatment goals (one question), diet (three questions), hypoglycemia (three questions), complications (seven questions), and insulin-specific knowledge (three questions). Scores were calculated by giving 1 point for a correct answer and 0 points for an incorrect answer, and the total score ranged from 0 to 23, with higher scores indicating higher levels of diabetes knowledge. The internal consistency of the dichotomous choices was KR-20 (Kuder–Richardson formula) = 0.74 when the reliability of the tool was tested at the time of tool development. In this study, KR-20 (Kuder–Richardson formula) was 0.65.

#### 2.2.3. Attitudes Toward Internet Health Information

This study assessed attitudes toward internet health information using a tool modified by Kim et al. [21] based on the ‘Internet health information-seeking model’ for adults that was originally developed by Noh et al. [22]. The tool consisted of 12 questions in four domains including perceived usefulness (three questions), perceived ease of use (three questions), information credibility (three questions), and information usefulness (three questions) regarding health information on the internet. The tool scored on a 5-point Likert scale ranging from 1 for “Not at all” to 5 for “Absolutely”, with higher scores indicating more positive attitudes toward internet health information. At the time of tool development, the reliability of the subscales was Cronbach’s α = 0.75–0.85 in the study by Noh et al. [21] and Cronbach’s α = 0.86 in this study.

#### 2.2.4. eHL

eHL refers to the ability to seek, explore, understand, and evaluate health information on the internet and the capacity to apply and disseminate the acquired knowledge to address and solve health problems [26]. This study used the eHL tool developed by Lee [36] based on the health literacy components described by Nutbeam [37]. The tool had 31 questions consisting of three subdomains: communicative eHL (11 questions), critical eHL (12 questions), and functional eHL (8 questions). Each question was scored on a 5-point Likert scale ranging from 5 for “Absolutely” to 1 for “Not at all”, with higher scores indicating higher levels of eHL. At the time of tool development, the reliability of the subscales of the tool was shown to range from Cronbach’s α = 0.90 to 0.92 in a study by Lee [35]. The reliability in this study was Cronbach’s α = 0.96 for the overall domain, the functional eHL Cronbach’s α = 0.93, communicative eHL Cronbach’s α = 0.94, and critical eHL Cronbach’s α = 0.94.

### 2.3. Data Collection

This study collected data through convenience sampling based on inclusion criteria from outpatients diagnosed with diabetes at a university hospital in rural South Korea over a 2-month period, from 14 January to 29 February 2024. In a separate room where privacy was secured, the purpose of the study was fully explained to the participants by the researcher one-on-one, and informed consent was obtained. Data were then collected using a structured questionnaire.

### 2.4. Data Analysis

The collected data were analyzed using IBM SPSS software version 27.0 for Windows (IBM Corp., Armonk, NY, USA). The general and disease-related characteristics of patients and the levels of main variables were analyzed using descriptive statistics such as mean, standard deviation, frequency, and percentage according to the measurements. After a normality check based on skewness and kurtosis, differences in eHL according to patients’ general and disease-related characteristics were analyzed using an independent *t*-test or one-way analysis of variance, with statistical significance set at *p* < 0.05. The correlation between the main variables was analyzed using Pearson’s correlation coefficient. The factors affecting eHL were identified using a hierarchical regression.

### 2.5. Study Ethics

This study was approved by the Institutional Review Board of D University WISE (DGU-IRB-20230020) before collecting data. Participants were informed orally and in writing about the purpose and method of the study, anonymity of participation, voluntary consent, and refusal to participate in the study, the right to withdraw, and possible benefits and disadvantages, and submitted the informed consent form.

## 3. Results

### 3.1. Differences in eHL by General Characteristics

Differences in eHL by general characteristics are presented in Table 1. Among 185 participants included in this study, males accounted for 69.2% (n = 128), compared to 30.8% (n = 57) for females, with a mean age of 55.60 (±6.77) years for all participants. In terms of education, 50.8% (n = 94) graduated from middle or high school. Briefly, 81.6% (n = 151) were married, and 81.1% (n = 150) had a job. eHL was significantly different by age (t = 6.12, *p* = 0.003), education (t = 5.50, *p* = 0.005), and alcohol consumption (t = −2.93, *p* = 0.004).

### 3.2. Differences in eHL by Disease-Related Characteristics

Differences in eHL by disease-related characteristics are presented in Table 2. The mean time since diagnosis was 115.42 (±88.78) months. High proportions of participants had non-diabetic chronic complications (75.1%, n = 139), had not received diabetes management education (73.0%, n = 135), had never used a health-related app or wearable device (67%, n = 124), and reported their subjective health status as fair (68.6%, n = 127). However, eHL differed according to experience using apps or wearable devices for health management (t = 2.05, *p* = 0.042).

### 3.3. Levels of Diabetes Knowledge, Attitudes Toward Internet Health Information, and eHL

The study variables and factor levels for this study are shown in Table 3. Patients’ diabetes knowledge scored a mean of 15.26 (±3.23) out of 23 points from 23 questions. Attitude toward internet health information was 3.19 (±0.57) out of 5 points. The scores were 3.16 (±0.67) out of 5 for eHL, 3.58 (±0.73) for functional eHL, 2.85 (±0.86) for communicative eHL, and 3.16 (±0.74) for critical eHL.

### 3.4. Associations Among Diabetes Knowledge, Attitudes Toward Internet Health Information and eHL

The results of the correlation analysis among the study variables are shown in Table 4. The correlation coefficients ranged from 0.25 to 0.62. eHL was positively correlated with diabetes knowledge (*r* = 0.31, *p* < 0.001) and attitudes toward internet health information (*r* = 0.62, *p* < 0.001). Diabetes knowledge also had a significantly positive correlation with attitudes toward internet health information (*r* = 0.25, *p* < 0.001).

### 3.5. Factors Affecting eHL

To identify the variables affecting eHL in middle-aged patients with diabetes, a two-stage hierarchical regression analysis was conducted (Table 5). Before hierarchical regression, the variance inflation factor (VIF) was determined to check for multicollinearity among variables, which revealed that the VIF was less than 10, indicating no multicollinearity. The Durbin–Watson statistic for the mutual independence of the residuals was 2.13, which was close to 2, indicating that the residuals were mutually independent.

In the hierarchical regression model 1, the variables showing significance in the univariate analysis (namely, age, education, alcohol consumption, and electronic device use) were entered after processing them as dummy variables. In the results, age (β = 0.29, *p* = 0.038) and alcohol consumption (β = 0.24, *p* = 0.040) were found to be significant, with an explanatory power of 10% (F = 4.23, *p* = 0.001).

In stage 2 of the hierarchical regression analysis, diabetes knowledge and attitudes toward internet health information were added to the stage 1 model. This increased the explanatory power of eHL to 42% (F = 17.46, *p* < 0.001) after adding the independent variables in stage 2. Attitudes toward internet health information (β = 0.55, *p* < 0.001), diabetes knowledge (β = 0.13, *p* = 0.040), and alcohol consumption (β = 0.12, *p* = 0.044) were identified as significant influencing factors on eHL.

## 4. Discussion

In the middle-aged population, who has recently experienced a high rate of diagnosis from pre-diabetes to diabetes, this study was conducted to identify the relationship between diabetes knowledge and attitudes toward internet health information and eHL to ultimately establish nursing intervention strategies to promote their health management behaviors.

This study identified attitudes toward internet health information, diabetes knowledge, and alcohol consumption as influential factors on eHL, which are further discussed below. The level of the eHL of participants in this study was 3.16 (±0.67) out of 5, which was lower than the level of 3.82 (±0.62) reported in a study that measured the level of the eHL of middle-aged adults using the same tool [38]. This difference likely resulted from the higher average age and lower education levels of the participants in this study. While the mean age of the patients in this study was 55.60 (±6.77) years old, their mean age in the previous study [38] was 46.04 (±5.45) years old, showing a slightly higher mean age of our participants. Moreover, the educational level of the patients in this study was at or below the middle and high school graduation level, accounting for 50.8%, while 79.6% of the patients in the previous study [38] were university graduates or higher. This is consistent with previous research that found eHL to be higher with younger age [39] and higher education [40]. Despite the lack of studies using the same tool making direct comparisons difficult, Yuan et al. [41] examined the association between online health information usage habits and eHL in 1061 adults aged 45–89 years in China’s middle-aged and elderly population and found that younger age was associated with higher levels of eHL, which is consistent with our findings. In addition, the low level of eHL in this study may be attributable to the location of the study site in a rural area where it is difficult to access various information on digital health and the fact that we included patients who were concentrated in the elderly population with low access and acceptance of digital health. Therefore, it is necessary to develop a strategy to increase the accessibility of digital health information and digital education environments both in rural areas and urban centers to enable people of all age groups to adapt to digital health information in the rapidly changing 4th revolution era. In the future, we expect to design customized education programs that consider age, education level, and experience using the digital devices of rural residents. In particular, for middle-aged people in their 30s, 40s, or 50s, we provide opportunities for residents to directly participate and learn by hosting community-based workshops and seminars. Furthermore, access to digital devices and the Internet may be limited in rural areas. Thus, we provide computers and tablets in public spaces as well as free Wi-Fi so that residents can access the Internet at any time.

This study identified attitudes toward internet health information as the most influential factor on eHL. In this study, more positive attitudes toward internet health information were associated with higher eHL levels. A study by Yang et al. [25], comparing the influencing factors of eHL in 397 young and older Koreans, also found attitudes toward internet health information to be a significant predictor, which was consistent with the results of this study. In a study by Liu et al. [42], examining the influencing factors of digital health literacy in 572 older Chinese adults, attitudes toward internet health information were also a significant predictor, which was also similar to the results of this study. In a study that examined factors affecting eHL among 423 chronically ill patients in low-income countries, Shiferaw et al. [43] found that more positive attitudes toward the use of online health resources were associated with higher levels of eHL, supporting the results of this study. Thus, it is necessary to develop intervention programs that promote positive perceptions of internet health information and facilitate the use of reliable, high-quality internet health information to increase perceived usefulness and perceived ease of use among middle-aged patients with diabetes.

The present study showed that diabetes knowledge affects eHL in middle-aged patients with diabetes. Although it is difficult to make direct comparisons due to a small number of studies examining the relationship between diabetes knowledge and eHL, Stellefson et al. [44] investigated factors affecting eHL among 176 patients with chronic obstructive pulmonary disease (COPD) aged 40 years and older in the United States and then discovered that knowledge related to COPD was a statistically significant predictor, with higher levels of knowledge related to COPD being associated with higher levels of eHL. Additionally, a previous study reported a positive association between knowledge of cervical cancer or human papilloma and eHL [45]. Moreover, the diabetes knowledge assessment in this study revealed that the subfactor with the highest correct response rate was the question about the therapeutic goals of diabetes, whereas the lowest correct response rate was found for diet. The highest rate of correct responses was for knowledge of treatment goals, which may be because the mean duration of diabetes in this study was 9.62 years, with 63.8% of participants having long-term diabetes of 5 years or more, and 75.1% having non-diabetic chronic complications, which probably provided them with more opportunities to learn about disease-related health markers such as blood pressure and target levels for blood glucose. On the other hand, dietary knowledge had the lowest correct answer rate, which may be because the study site was a small university hospital with 300 beds, lacking nutrition specialists and nutrition education, and a large population of older adults lived in the area, making it difficult to obtain healthy dietary information.

Alcohol consumption was identified as a demographic factor influencing eHL in this study. Despite using a different age group, a study that examined the effectiveness of an e-health educational intervention among 2939 Chinese college students also found higher levels of eHL among the alcohol-drinking group [46]. In this study, 81.1% (n = 150) of patients were self-employed or workers, with a mean age of 55.60 (±6.77) years and with 69.1% being males. In South Korea, there is a culture of generously accepting alcohol consumption, and those with higher socioeconomic status and males are more likely to be frequent drinkers [47]. In addition, middle-aged people are at the peak of their work activities and are active in economic and social activities [6]. Accordingly, there are more frequent drinking opportunities as a social activity [48], which may have led to the exchange of information. Another study examined the association between social capital and eHL among 4257 local community-dwelling elderly people and found that stronger social connections enhancing social interaction were associated with higher levels of eHL [49]. In contrast, some previous studies reported that eHL had no statistically significant association with drinking status [50,51]. Thus, replications of the results are needed to confirm the clear association between drinking as a social activity and eHL.

This study has some limitations. First, this was a descriptive survey study that included targeting middle-aged people who were patients with diabetes and who received outpatient care at a single university hospital. This study has some limitations. First, this was a descriptive survey study that targeted middle-aged people (40–64 years old) with diabetes who received outpatient care at a single university hospital. Therefore, the generalizability of our study findings is limited, and future multicenter, all-age groups studies should be conducted. Second, this study was a cross-sectional survey study, and the data were collected using convenience sampling making it difficult to establish a causal relationship between the variables. Thus, a randomized controlled trial excluding bias is needed for further investigation. Finally, this study was measured by distributing subjective questionnaires, which may be prone to error; thus, the results should be interpreted with caution.

Nevertheless, we believe that this study is significant because it identified the association among eHL, diabetes knowledge, and attitudes toward internet health information in middle-aged patients with diabetes who are at the most productive and economically active stage of the life cycle, providing evidence for the importance of managing chronic disease such as diabetes potentially rapidly progressive disease. It may play a role in promoting health management based on correct information by raising awareness of the importance of knowledge about diseases and attitudes toward searching for information via the Internet.

### Reproducibility of the Study

- This study was designed as a descriptive correlational study to examine the relationship between e-health literacy (eHL), diabetes knowledge, and attitudes toward internet health information in middle-aged patients with diabetes. To ensure reproducibility, the following methodological considerations were applied.
- Standardized Data Collection Instruments: Validated self-report structured questionnaires were used to measure eHL, diabetes knowledge, and attitudes toward internet health information. The same versions of the questionnaires were administered to all participants to maintain consistency.
- Defined Study Population and Setting: The study population included 185 middle-aged patients with diabetes receiving follow-up care at a university hospital in South Korea. Clearly defined inclusion and exclusion criteria ensured a homogeneous sample, facilitating reproducibility in similar clinical settings.
- Statistical Analysis and Transparency: Data were analyzed using IBM SPSS 27.0, a widely recognized statistical software, ensuring that other researchers can replicate the analytical approach.
- Temporal and Contextual Considerations: The study was conducted between 14 January and 29 February 2024, providing a clear timeframe for data collection. While the findings may be specific to context of South Korea, the methodology can be adapted for use in different populations with similar demographic and healthcare backgrounds.
- Potential for Replication: Researchers seeking to replicate this study should use similar patient populations, validated questionnaires, and statistical methods to ensure comparable findings. Future studies may consider longitudinal designs to assess causality or expand the study to different healthcare settings for broader generalizability. By maintaining methodological rigor and transparency, this study provides a foundation for reproducibility, allowing future research to validate and expand upon its findings.

## 5. Conclusions

A higher eHL in middle-aged patients with diabetes is associated with higher attitudes toward internet health information, higher diabetes knowledge, and higher levels of alcohol consumption. Of these, attitudes toward internet health information is the most influencing factor. The incidence of chronic diseases increases in middle age (40–64 years old) compared to other age groups, so in order to prepare for a healthy old age, eHL, which is the correct selection of information along with attitude toward information in the midst of a flood of correct information, is considered more important. Therefore, it is necessary to develop intervention strategies to promote eHL, which is the ability to explore, evaluate, and share their own health information in middle-aged patients with diabetes, thereby cultivating self-management skills to manage diabetes, a chronic disease. Additionally, healthcare providers should offer ongoing training and monitoring to ensure that the patients acquire and utilize the correct information.

## Figures and Tables

**Table 1:** Differences in e-health literacy by general characteristics (n = 185).

Variables	Categories	n (%) or Mean ± SD	eHealth Literacy	
			Mean ± SD	t or F (*p*) Scheffé Test
Age ^†^	40–49 ^a^	39 (21.1)	3.41 ± 0.61	6.12 (0.003) a > c
(years)	50–59 ^b^	81 (43.8)	3.20 ± 0.58	
	60–64 ^c^	65 (35.1)	2.96 ± 0.76	
		55.60 ± 6.77	3.16 ± 0.67	
Sex	Male	128 (69.2)	3.16 ± 0.66	−0.15 (0.882)
	Female	57 (30.8)	3.15 ± 0.70	
Educational level ^†^	≤Elementary ^a^	7 (3.8)	3.00 ± 0.85	5.50 (0.005) c > b
	Middle to high school ^b^	94 (50.8)	3.01 ± 0.69	
	≥College ^c^	84 (45.4)	3.33 ± 0.60	
Marital status	Unmarried	18 (9.7)	3.12 ± 0.74	1.31 (0.269)
	Married	151 (81.6)	3.18 ± 0.66	
	Divorced	9 (4.9)	3.06 ± 0.70	
	Widowed	3 (1.6)	2.35 ± 1.06	
	Others	4 (2.2)	3.40 ± 0.26	
Employment status	Yes	150 (81.1)	3.19 ± 0.67	1.17 (0.244)
	No	35 (18.9)	3.04 ± 0.71	
Alcohol drinking	Yes	95 (51.4)	3.28 ± 0.60	−2.93 (0.004)
	No	90 (48.6)	3.00 ± 0.71	
Smoking status	Current smoker	50 (27.0)	3.16 ± 0.67	0.76 (0.469)
	Ex-smoker	4323.2)	3.22 ± 0.68	
	Non-smoker	92 (49.7)	3.18 ± 0.66	

SD = standard deviation. ^†^ Post hoc test was used with the Scheffé test. ^a–c^ Different superscript letters indicate significant differences between groups (*p* < 0.05, by Scheffé’s post-hoc test).

**Table 2:** Differences in e-health literacy by disease-related characteristics (n = 185).

Variables	Categories	n (%) or Mean ± SD	eHealth Literacy	
			Mean ± SD	t or F (*p)*
Diagnosis time (months)	3–12	13 (7.0)	2.88 ± 0.71	1.16 (0.327)
	13–60	54 (29.2)	3.18 ± 0.67	
	61–120	57 (30.8)	3.24 ± 0.56	
	≥121	61 (33.0	3.11 ± 0.67	
		115.42 ± 88.78	3.16 ± 0.67	
Non-diabetic chronic complications	Yes	139 (75.1)	3.17 ± 0.67	0.38 (0.705)
	No	46 (24.7)	3.12 ± 0.69	
Diabetes management education	Yes	50 (27.0)	3.14 ± 0.69	−0.22 (0.824)
	No	135 (73.0)	3.16 ± 0.67	
Experience in using electronic health devices	Yes	61 (33.0)	3.30 ± 0.60	2.05 (0.042)
	No	124 (67.0)	3.09 ± 0.70	
Subjective health status	Poor	38 (20.5)	3.20 ± 0.72	0.72 (0.930)
	Moderate	127 (68.6)	3.15 ± 0.66	
	Good	20 (10.8)	3.15 ± 0.70	

SD = standard deviation.

**Table 3:** Levels of diabetes knowledge, attitudes toward internet health information, and e-health literacy (n = 185).

Variables	Categories	Item	Mean ± SD	Accuracy (%)	Range
					Min–Max
Diabetes knowledge		23	15.26 ± 3.23		6–22
	General knowledge	6	4.26 ± 1.27	71%	
	Treatment goals	1	0.81 ± 0.39	81%	
	Dietary therapy	3	1.35 ± 0.83	45%	
	Hypoglycemia	3	1.73 ± 0.64	57%	
	Complications	7	5.29 ± 1.39	75%	
	Knowledge on insulin	3	1.81 ± 0.83	60%	
Attitudes toward internet health information		12	3.19 ± 0.57		1.83–4.58
	Perceived usefulness	3	3.45 ± 0.64		
	Perceived ease of use	3	3.59 ± 0.67		
	Information reliability	3	3.00 ± 0.67		
	Information utilization	3	2.73 ± 0.91		
E-health literacy		31	3.16 ± 0.67		1.32–4.74
	Functional eHL	8	3.58 ± 0.73		
	Communicative eHL	11	2.85 ± 0.86		
	Critical eHL	12	3.16 ± 0.74		

SD = standard deviation; eHL = e-health literacy; Min = Minimum; Max = Maximum.

**Table 4:** Associations among diabetes knowledge, attitudes toward internet health information, and e-health literacy (n = 185).

Variables	Diabetes Knowledge	Attitudes Toward Internet Health Information	eHealth Literacy
	*r* (*p*)	*r* (*p*)	*r* (*p*)
Diabetes knowledge	1		
Attitudes toward internet health information	0.25 (<0.001)	1	
E-health literacy	0.31 (<0.001)	0.62 (<0.001)	1

**Table 5:** Factors affecting e-health literacy (n = 185).

Variables	Model 1				Model 2			
	B	SE	β	t (*p)*	B	SE	β	t (*p)*
Age (ref: 60–64 years ^a^)	0.29	0.14	0.18	2.09 (0.038)	0.14	0.11	0.08	1.22 (0.222)
Educational level (ref: ≤elementary ^a^)	0.24	0.26	0.18	0.93 (0.356)	0.12	0.21	0.09	0.55 (0.580)
Alcohol drinking (ref: no ^a^)	0.24	0.10	0.15	2.07 (0.040)	0.15	0.08	0.12	2.02 (0.044)
Experience in using electronic devices (ref: no ^a^)	0.15	0.10	0.10	1.46 (0.146)	0.07	0.08	0.04	0.64 (0.526)
Diabetes knowledge					0.03	0.01	0.13	2.07 (0.040)
Attitudes toward internet health information					0.64	0.07	0.55	9.14 (<0.001)
	F = 4.23, *p* = 0.001 (Adj. R^2^ = 0.10)				F = 17.46, *p* < 0.001 (Adj. R^2^ = 0.42)			

SE = standard error, ref = reference, Adj = adjusted. Each β represents a standardized coefficient; each B represents an unstandardized coefficient. ^a^ Reference groups of dummy variables were age (40–49 and 50–59 years), educational level (middle to high school, ≥college), alcohol drinking (yes), and experience in using electronic devices (yes).

## Data Availability

Data are available from the authors upon request owing to privacy or ethical restrictions.

## Abbreviations

The following abbreviations are used in this manuscript:

COPD	chronic obstructive pulmonary disease
eHL	e-health literacy
VIF	variance inflation factor
